# STABILITY AND INTER-RELATIONSHIPS OF INFLAMMATORY MARKERS ACROSS TWO WEEKS IN OLDER ADULTS

**DOI:** 10.1093/geroni/igad104.3600

**Published:** 2023-12-21

**Authors:** Robert Tennyson, Molly Wright, Erik Knight, Jennifer Graham-Engeland, Christopher Engeland

**Affiliations:** Pennsylvania State University, University Park, Pennsylvania, United States; Pennsylvania State University, University Park, Pennsylvania, United States; University of Colorado Boulder, Boulder, Colorado, United States; Pennsylvania State University, University Park, Pennsylvania, United States; Pennsylvania State University, University Park, Pennsylvania, United States

## Abstract

The temporal stability of basal and stimulated inflammatory cytokines is unclear in older adults, despite often being used to characterize inflammatory status. Further, it is not apparent how the stability of these markers and their multi-dimensional relationships connect to healthy aging. This analysis examines these issues in 227 participants from the Einstein Aging Study [ages: 70-90 (Mean=76.7), 67% women] with blood draws ~2 weeks apart. Temporal stability was assessed by intra-class coefficients (ICCs) obtained from linear mixed models (controlling for days between measurements, age, gender) for a panel of basal and lipopolysaccharide (LPS)-stimulated cytokines (interleukin [IL]-1β, IL-4, IL-6, IL-8, IL-10, tumor necrosis factor [TNF]-α). Sensitivity analyses compared ICCs across genders and individuals reporting high vs. low subjective health. A composite measure of basal cytokines exhibited strong reliability (ICC: 0.854), whereas individual basal cytokines exhibited moderate (>0.5) to very high reliability (>0.9). Conversely, a composite measure of stimulated cytokines demonstrated moderate reliability (ICC: 0.552), with individual stimulated cytokines having only low (< 0.5) to moderate reliability. No appreciable differences were observed by gender, but individuals reporting low (ICC: 0.457) vs. high (ICC: 0.631) subjective health exhibited considerably lower reliability for the stimulated composite measure. In sum, basal cytokines had moderate to high stability, whereas stimulated cytokines exhibited relatively low stability. Given that individuals reporting lower subjective health demonstrated greater variability in stimulated cytokines, higher fluctuations in inflammatory responsivity may reflect suboptimal health in older adults. The inter-relationships of these cytokines, explored via principal component analysis, will also be discussed.

